# Diffuse normolipemic plane xanthoma and hepatitis C: chance?^☆^^☆☆^

**DOI:** 10.1016/j.abd.2019.08.026

**Published:** 2020-02-17

**Authors:** Maria Carolina Casa Souza, Paulo Henrique Teixeira Martins, Analú Vivian, Laura Luzzatto

**Affiliations:** Department of Dermatology, Santa Casa de Misericórdia de Porto Alegre, Porto Alegre, RS, Brazil

Dear Editor,

Diffuse Normolipemic Plane Xanthoma (DNPX) is a rare acquired dermatosis, clinically characterized by patches and/or yellow-orange plaques symmetrically distributed. It is an uncommon type of non-Langerhans histiocytosis that occurs due to the deposition of lipids in the skin and, in almost half of cases, occur in the absence of hypercholesterolemia.^1^ DNPX has been associated with systemic diseases such as multiple myeloma and other hematological and lymphoproliferative neoplasms.^2^ A case of diffuse normolipemic plane xanthoma with no lipid profile changes is reported in a patient with a recent diagnosis of Hepatitis C.

A 61 year-old man with a history of onset of asymptomatic yellowish spots, 2 years ago, initially on the eyelids. In one year, the lesions also appeared in the armpits, in the inguinal, genital and gluteal regions. He had a recent diagnosis of Hepatitis C (HCV), without treatment. He denied other comorbidities and use of medications. On examination he had yellowish plaques with a symmetrical distribution in the periorbital region bilaterally (Fig. 1), and plaques with regular, well-delimited borders, yellow-orange in the armpits (Fig. 2), scrotal region, inguinal, gluteal, and also in the thighs. Laboratory tests – blood count, blood glucose, renal, thyroid, protein gram and liver function – were normal and the lipid profile showed no alterations. After anatomopathological examination, a cluster of foamy histiocytes was observed in the superficial dermis between the collagen fibers (Fig. 3), concluding the diagnosis of plane xanthoma. The patient is followed up and under surveillance for the appearance of other associated diseases (such as monoclonal gammopathies), although totally asymptomatic.

**Figure 1:**
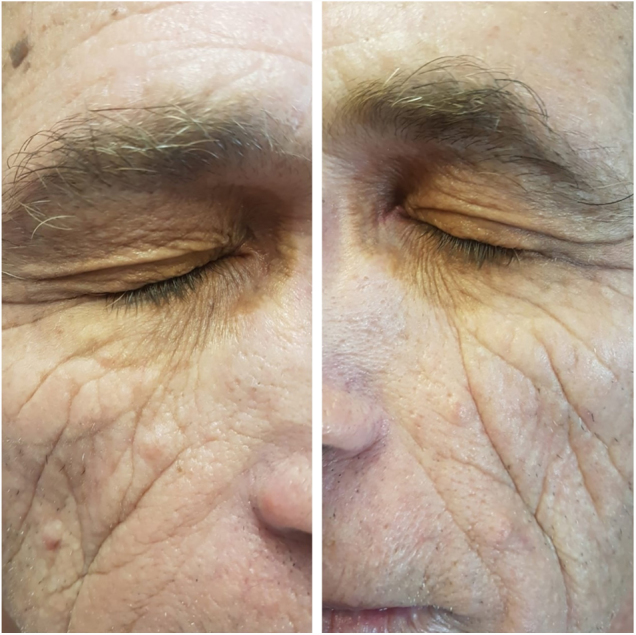
Yellowish, well delimited plaques in the region of eyelids bilaterally.

**Figure 2:**
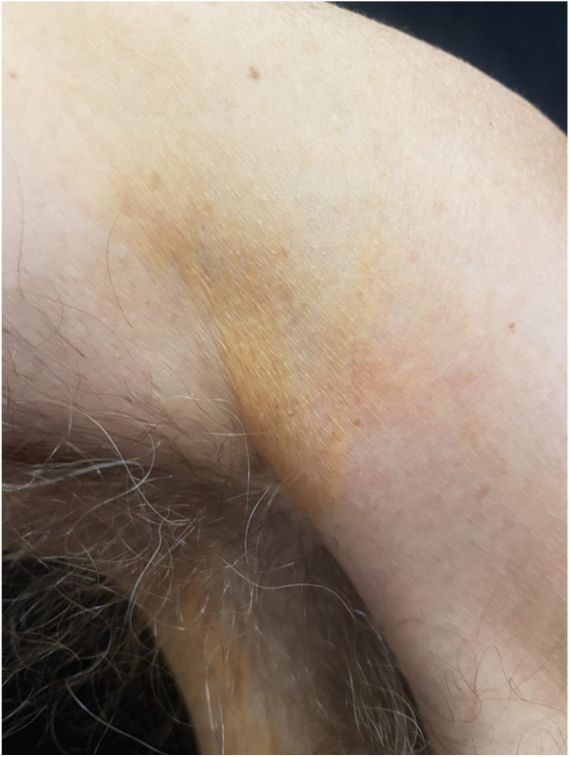
Orange yellow plaque in axillary region.

**Figure 3:**
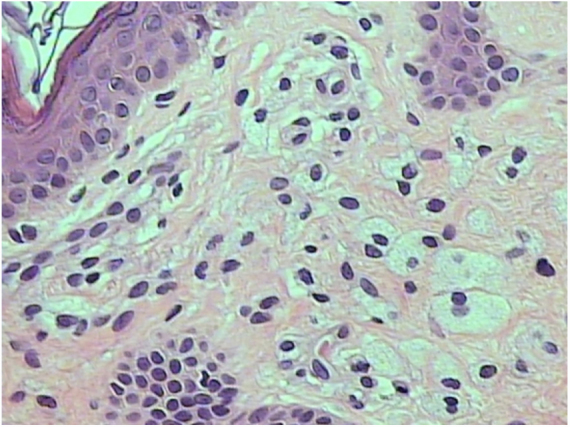
Aggregation of foamy histiocytes in the superficial dermis between collagen fibers.

DNPX is a rare type of non-Langerhans histiocytosis that usually appears initially with a palpebral xanthelasma, as is the case, with later appearance flesions usually in the neck, trunk, upper dorsum, cubital fossae, and extremities. These distribution of xanthomatous lesions in DNPX is unique, although it varies among cases.^3^ In the histopathology, it presents with foamy cells – macrophages with lipid droplets – and variable number of giant Touton cells, lymphocytes and foamy histiocytes can be seen in the upper dermis.^1^ No standardized treatment is available yet, but there are papers citing treatments with ablative lasers, such as the erbium-YAG4 laser.^4^

Hepatitis C is a systemic disease that can cause manifestations in various organs and systems. It is estimated that approximately 74% of patients experience at least one extra hepatic manifestation of the disease during their lifetime, with 17% of them having dermatological manifestations. The most commonly associated dermatoses are cryoglobulinemia, lichen planus, porphyria cutanea tarda and acral necrolytic erythema. It is believed that most of these diseases present as path physiology the formation and deposition of immune complexes in the tissues, although the mechanism of these diseases is not completely understood. Other dermatological diseases also have been shown to be associated with hepatitis C, but with less consistency, such as cutaneous B-cell lymphoma, erythema multiforme, leukocytoclastic vasculitis and urticaria.^5^

XPDN has been associated with hematologic disorders, particularly multiple myeloma and monoclonal gammopathy; however, leukemia, lymphoma and Castleman's disease have also been associated with the disease.^2^, ^3^ The patient in the case did not present any evidence of hematologic disorder at the time, despite being investigated. In the present XPDN case, the presence of the HCV infection was verified, which may be a coincidence; but it is important to emphasize the immunogenic importance of HCV, as it is a possible trigger of some dermatological conditions.

## Financial support

None declared.

## Authors’ contributions

Maria Carolina Casa Souza: Approval of the final version of the manuscript; conception and planning of the study; elaboration and writing of the manuscript; obtaining, analysis, and interpretation of the data; effective participation in research orientation; intellectual participation in the propaedeutic and/or therapeutic conduct of the studied cases; review of the literature; critical review of the manuscript.

Paulo Henrique Teixeira Martins: Statistic analysis; approval of the final version of the manuscript; conception and planning of the study; elaboration and writing of the manuscript; obtaining, analysis, and interpretation of the data; effective participation in research orientation; intellectual participation in the propaedeutic and/or therapeutic conduct of the studied cases; review of the literature; critical review of the manuscript.

Analú Vivian: Approval of the final version of the manuscript; conception and planning of the study; elaboration and writing of the manuscript; effective participation in research orientation; intellectual participation in the propaedeutic and/or therapeutic conduct of the studied cases; review of the literature; critical review of the manuscript.

Laura Luzzatto: Obtaining, analysis, and interpretation of the data; effective participation in research orientation.

## Conflicts of interest

None declared.
